# A novel sensitive detection method for DNA methylation in circulating free DNA of pancreatic cancer

**DOI:** 10.1371/journal.pone.0233782

**Published:** 2020-06-10

**Authors:** Keiko Shinjo, Kazuo Hara, Genta Nagae, Takayoshi Umeda, Keisuke Katsushima, Miho Suzuki, Yoshiteru Murofushi, Yuta Umezu, Ichiro Takeuchi, Satoru Takahashi, Yusuke Okuno, Keitaro Matsuo, Hidemi Ito, Shoji Tajima, Hiroyuki Aburatani, Kenji Yamao, Yutaka Kondo

**Affiliations:** 1 Division of Cancer Biology, Nagoya University Graduate School of Medicine, Nagoya, Japan; 2 Department of Gastroenterology, Aichi Cancer Center Hospital, Nagoya, Japan; 3 Genome Science Laboratory, Research Center for Advanced Science and Technology, The University of Tokyo, Tokyo, Japan; 4 Department of Computer Science, Nagoya Institute of Technology, Nagoya, Japan; 5 School of Information and Data Sciences, Nagasaki University, Nagasaki, Japan; 6 RIKEN Center for Advanced Intelligence Project, Tokyo, Japan; 7 Department of Experimental Pathology and Tumor Biology, Nagoya City University Graduate School of Medical Sciences, Nagoya, Japan; 8 Center for Advanced Medicine and Clinical Research, Nagoya University Hospital, Nagoya, Japan; 9 Division of Cancer Epidemiology and Prevention, Aichi Cancer Center Research Institute, Nagoya, Japan; 10 Department of Cancer Epidemiology, Nagoya University Graduate School of Medicine, Nagoya, Japan; 11 Division of Cancer Information and Control, Aichi Cancer Center Research Institute, Nagoya, Japan; 12 Department of Descriptive Cancer Epidemiology, Nagoya University Graduate School of Medicine, Nagoya, Japan; 13 Laboratory of Epigenetics, Institute for Protein Research, Osaka University, Osaka, Japan; 14 Department of Gastroenterology, Narita Memorial Hospital, Toyohashi, Japan; Sapporo Ika Daigaku, JAPAN

## Abstract

Despite recent advances in clinical treatment, pancreatic cancer remains a highly lethal malignancy. In order to improve the survival rate of patients with pancreatic cancer, the development of non-invasive diagnostic methods using effective biomarkers is urgently needed. Here, we developed a highly sensitive method to detect DNA methylation in cell-free (cf)DNA samples based on the enrichment of methyl-CpG binding (MBD) protein coupled with a digital PCR method (MBD–ddPCR). Five DNA methylation markers for the diagnosis of pancreatic cancer were identified through DNA methylation microarray analysis in 37 pancreatic cancers. The sensitivity and specificity of the five markers were validated in another independent cohort of pancreatic cancers (100% and 100%, respectively; n = 46) as well as in The Cancer Genome Atlas data set (96% and 90%, respectively; n = 137). MBD–ddPCR analysis revealed that DNA methylation in at least one of the five markers was detected in 23 (49%) samples of cfDNA from 47 patients with pancreatic cancer. Further, a combination of DNA methylation markers and the *KRAS* mutation status improved the diagnostic capability of this method (sensitivity and specificity, 68% and 86%, respectively). Genome-wide MBD-sequencing analysis in cancer tissues and corresponding cfDNA revealed that more than 80% of methylated regions were overlapping; DNA methylation profiles of cancerous tissues and cfDNA significantly correlated with each other (*R* = 0.97). Our data indicate that newly developed MBD–ddPCR is a sensitive method to detect cfDNA methylation and that using five marker genes plus *KRAS* mutations may be useful for the detection of pancreatic cancers.

## Introduction

Pancreatic cancer is the fourth leading cause of cancer deaths, of which the five-year survival rate is less than 5%, with the number of cases predicted to increase worldwide [1]. Its poor prognosis is due to several complex factors; however, one crucial reason is that the majority of patients are diagnosed at a later stage, when only about 20% of patients are eligible for surgical resection [2]. Serum carbohydrate antigen 19–9 (CA19-9) is a classical biomarker that is widely used for detecting pancreatic cancer. However, it is inadequate because of its low sensitivity (41–86%) and specificity (33–100%) [2]. Therefore, the development of more efficient and reliable biomarkers to diagnose pancreatic cancer at an operable stage is urgently required.

Tumors release fragments of cell-free nucleic acids that include cell-free DNA (cfDNA), as well as mRNA and micro(mi)RNAs into the bloodstream, and which are considered as potential biomarkers for a cancer diagnosis. Detecting tumor-specific nucleic acids with genetic and epigenetic alterations, such as mutations, copy number changes, and DNA methylation, enables us to diagnose and monitor tumors using what is called a “liquid biopsy” [3–5]. Epigenetic mechanisms, including DNA methylation, histone modification, and chromatin remodeling, have been thought to be closely associated with gene expression and reflect tumor phenotypes [6]. Of these, DNA methylation markers are considered particularly powerful diagnostic tools in the clinic due to their stable nature. A DNA methylation assay for *SEPT9* in serum has been initially approved by the Food and Drug Administration and used for improving colorectal cancer screening [7, 8]. With regard to pancreatic cancer, DNA methylation markers, cyclin-dependent kinase inhibitor 2A (*CDKN2A*/*p16*), ras-associated domain family member 1 (*RASSF1A*) and neuronal pentraxin 2 (*NPTX2*) in cfDNA are considered diagnostic markers. However, none of these are sufficiently powerful in diagnosing pancreatic cancer since a relatively insensitive method (methylation-specific PCR) is used and/or these genes are also frequently found methylated in other types of cancers [9, 10].

Detection of DNA methylation in tissues and body fluids is based on two steps [11]. The first step is the distinguishing of methylated and unmethylated DNA fragments by either physical separation (e.g., methyl-binding protein) or by changing their sequence (e.g., bisulfite conversion treatment). The second step involves analyzing the fragments by PCR, microarray or DNA sequencing. Currently, because of the limited amount of cfDNA in blood samples and a reduction in sequence complexity after bisulfite conversion, which sometimes produces biased results due to sequence mismatching in certain genetic loci [12], more sensitive and accurate detection methods using novel technology are required.

In this study, in order to identify effective DNA methylation markers for the diagnosis of pancreatic cancer, we first examined DNA methylation markers using fine-needle aspiration (FNA) samples of pancreatic cancer and Illumina genome-wide DNA methylation array. Furthermore, we developed a highly sensitive method based on methyl-CpG binding (MBD) protein enrichment coupled with a droplet digital PCR method (MBD–ddPCR). Our approach provides evidence that the detection of aberrant DNA at certain loci in cfDNA using new technology may be a promising approach for the diagnosis of pancreatic cancer.

## Materials and methods

### DNA extraction from tissue and blood samples

Tumor tissue samples (n = 83) were collected by endoscopic ultrasound–guided fine-needle aspiration biopsy (FNA) at Aichi Cancer Center Hospital from August 2009 to July 2012. Chronic pancreatitis tissue samples (n = 6) and tissue from other benign pancreatic diseases (n = 6) were obtained from patients who underwent surgery at Nagoya City University Hospital. FNA samples were kept in RNAlater (Thermo Fisher Scientific, Waltham, MA, USA) and stored at -80°C. DNA was extracted from tissues using a DNeasy Blood & Tissue kit (Qiagen, Hilden, Germany). DNA from formalin-fixed paraffin-embedded (FFPE) tissues was extracted with a QIAamp DNA FFPE tissue kit (Qiagen). DNA from the normal pancreatic tissue (n = 5) was obtained from BioChain (Newark, CA, USA).

Blood samples were obtained from patients before they underwent endoscopic examination. Blood was also obtained from normal volunteers (n = 16) without a history of cancer who visited Aichi Cancer Center Hospital. Blood samples were centrifuged at 2,000×g for 10 min at room temperature and serum aliquots were stored at -80°C until use. Cell-free DNA (cfDNA) was extracted from 1 mL of serum with a QIAamp Circulating Nucleic Acid kit (Qiagen) according to the manufacturer’s protocol. The DNA concentration were determined with a Qubit 2.0 Fluorometer (Thermo Fisher Scientific). The length distribution of cfDNA was examined by Bioanalyzer 2100 (Agilent Technologies, Santa Clara, CA, USA).

All samples and clinical data were collected after appropriate institutional review board approval was received and written informed consent had been obtained from all the patients. FNA samples underwent histological evaluation and were diagnosed as pancreatic adenocarcinoma. This study received approval of the Ethics Committee of Aichi Cancer Center, the Ethics Committee of Nagoya City University, and the Ethics Committee of Nagoya University. All methods were performed in accordance with the relevant guidelines and regulations.

### DNA methylation analysis

Genomic DNA (500–1000 ng) was bisulfite converted using an Epitect Plus bisulfite kit (Qiagen). DNA methylation levels were measured by pyrosequencing (Qiagen) or real–time PCR [13] (Thermo Fisher Scientific). Primer sequences and PCR conditions are shown in S1 Table. Fully methylated DNA was prepared by treating genomic DNA with SssI methylase (New England Biolabs [NEB], Ipswich, MA, USA). Unmethylated DNA was prepared by GenomiPhi DNA amplification kit (GE Healthcare Life-Science, Chicago, Il, USA) according to the manufacturer’s protocol.

### Genome–wide DNA methylation analysis

**Fig 1:**
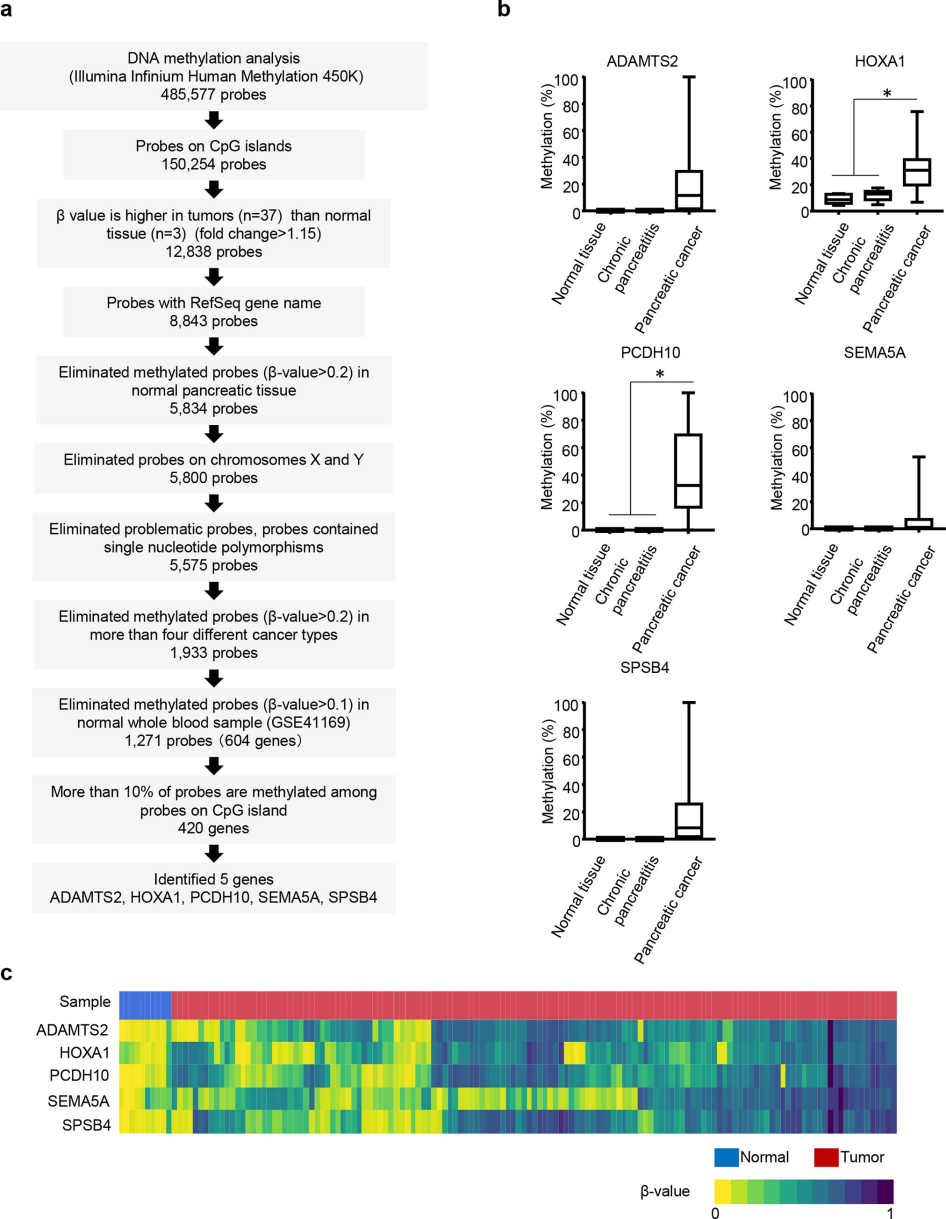
Methylation status of five marker genes in pancreatic cancer tissue. (a) Schema of how we identified five marker genes from methylation analysis of Illumina Infinium HM450. (b) Methylation level of five markers in normal (n = 5), chronic pancreatitis (n = 6), and pancreatic tumor (n = 22) tissues. DNA methylation levels of each gene are indicated on the y-axis. The median is indicated by a bold line inside the box whose ends denote the upper and lower quartiles. Error bars represent the 5 and 95 percentile values. *, *P*<0.05. (c) A heatmap of five methylation markers in The Cancer Genome Atlas (TCGA) sample, normal tissue (n = 10) and pancreatic tumor tissues (n = 137). Colors correspond to β-values as indicated (zero means a site is completely unmethylated while one means it is completely methylated). In the sample column, blue and red indicated normal and cancer tissues, respectively.

Bisulfite converted DNA was analyzed by Illumina Infinium Human Methylation 450K BeadChips (HM450; Illumina, San Diego, CA, USA) according to the manufacturer’s protocol. β-values were used for statistical analysis. From 485,577 Ilumina Infinium probes, we removed potentially problematic probes, those probes that contained single nucleotide polymorphisms, and probes on chromosomes X and Y. We subsequently selected 5,575 probes that were highly methylated in tumor samples compared to normal samples (fold change >1.15) and located in CpG islands. We further compared the DNA methylation status of 10 types of cancers (Pancreatic cancer [PADD, 148 cases]; lung adenocarcinoma [LUAD, 39 cases]; bladder urothelial carcinoma [BLCA, 44 cases]; colorectal cancer [CRC, 51 cases]; breast invasive carcinoma [BRCA, 50 cases]; renal cell carcinoma [KIRC, 40 cases]; acute myeloid leukemia [LAML, 40 cases]; lung squamous cell carcinoma [LUSC, 47 cases]; skin cutaneous melanoma [SKCM, 40 cases]; gastric adenocarcinoma [STAD, 29 cases]) and ten normal pancreatic tissues using HM450 DNA methylation data from the publicly available The Cancer Genome Atlas (TCGA) database (https://portal.gdc.cancer.gov/). Among the 5,575 probes, 3,642 probes were excluded, those mean β-values in each cancer type are over 0.2 in more than 4 types of cancers (i.e. methylated probes in multiple cancer types). Rest of the 1,933 probes, we analyzed DNA methylation data of normal whole blood (GSE41169) and excluded probes with β-values in normal blood of over 0.1. In the end, 1,271 probes (604 genes) were selected to be assessed for the diagnosis of pancreatic cancer. In order to select stably methylated genes, we pick up genes for which more than 10% of probes are methylated (420 genes; Fig 1A). We defined DNA methylated probes as those with a β-value >0.2, as reported previously [14]. To exclude slightly methylated probes in normal blood (GSE41169), probes with a β-value >0.1 were eliminated. The maximum β-value of probes was used to calculate the frequency of methylation (%) in each gene.

### *KRAS* mutation analysis

*KRAS* mutation status was analyzed by direct sequencing or pyrosequencing in tissue samples as reported previously [15, 16]. In blood samples, 3 to 10 ng of cfDNA was tested with a multiplex ddPCR KRAS screening kit (Bio-Rad, Pleasanton, CA, USA; G12A, G12C, G12D, G12R, G12S, G12V, and G13D) or multiplex ddPCR KRAS Q61 screening kit (Q61H, Q61K, Q61L, and Q61R) to distinguish wild type alleles from mutant alleles by a QX200 Droplet Digital PCR system (Bio-Rad). The lower limit of detection was set at 0.2% of mutant allele frequency [17].

### Methyl-CpG binding (MBD1)-ddPCR

DNA was incubated with 2.5 mg of His-GST–MBD protein [18] and 20 μL of Dynabeads Histag-isolation (Thermo Fisher Scientific) in MBD binding/wash buffer (100 mM Tris-HCl, pH 7.5, 1.6 M NaCl, 0.15% Tween 20) overnight at 4°C. Each time, 10 nM of control oligos, methylated and unmethylated sequences of yeast were added as positive and negative controls for MBD pull down (S1 Table). After incubation, the supernatant fraction that contained unmethylated DNA was removed. The beads were then washed four times with MBD binding/wash buffer. The beads were incubated with 100 μg of Proteinase K in elution buffer (50 mM Tris-HCl, pH 7.5, 10 mM EDTA, 0.5% SDS) at 50°C for 3 h. After incubation, AMpure XP beads (×1.8; Beckman Coulter, Indianapolis, IN, USA) were added to the purified methylated DNA and finally DNA was eluted with 0.1 × Tris-EDTA buffer (TE).

Chromatin end repair of the eluted DNA was performed by adding 5 μL of buffer (50 mM Tris-HCl, 10 mM MgCl_2_, 1 mM ATP, 10 mM dithiothreitol [DTT]), 20 mM dNTP, 2.5 μL of T4 polynucleotide kinase (10 U/μL, NEB), and 0.3 μL of T4 polymerase (3 U/μL, NEB). Samples were incubated at 12°C for 25 min, then 25°C for 25 min, followed by purification with AMpure XP beads (×1.8). Samples were then A-tailed by adding 10 μL of buffer (50 mM NaCl, 10 mM Tris-HCl, 10 mM MgCl_2_, 1 mM DTT), 10mM ATP, and Klenow (3’, 5’, -exo, 5 U/μL, NEB) and incubated at 37°C for 30 min. After incubation, DNA was purified with AMpure XP beads (×1.8). Then adaptors (S1 Table) were ligated to the end of DNA by adding 29 μL of Quick ligation buffer and 3 μL of Quick ligase (NEB). DNA was purified with AMpure XP beads (×1.8) and eluted with 18 μL of 0.1×TE. Finally, DNA was amplified with an adaptor sequence (S1 Table; KAPA HiFi HotStart ready mix; KAPA Biosystems, Wilmington, MA, USA). Amplified DNA was purified with AMpure XP beads (×1.8) [19]. The eluted DNA (20 μL) was used for ddPCR assays (1 μL for each assay).

All assays included 100% and 0% methylated DNA as controls. From the control samples, we acquired formulae. According to these formulae, the percentage methylation of each sample was calculated by the correction of copy numbers of the control sample.

### MBD1-seq

The aforementioned methylated DNA enriched by MBD1 protein was used to generate indexed libraries with an NEBNext Ultra II DNA Library Prep Kit for Illumina (NEB). The libraries were sequenced (75-bp paired-end) on a HiSeq 2500 system (Illumina). The reads were aligned to hg19/GRCh37 with BWA [20]. Duplicate reads and longer fragments (longer than 150bp) were removed. Methylation peaks were determined with HOMER software (v4.10, 5-16-2018; style histone, size 150, minDist 370) [21]. The correlation between samples was calculated by deepTools2 software [22]. An output bedGraph file was generated by HOMER and transformed to a binary format (TDF file) by igvtools for visualization in the Integrative Genome Viewer (IGV) [23].

### Statistical analysis

Statistical analysis and plotting of data were performed using GraphPad Prism 7 (GraphPad Software, San Diego, CA, USA) and JMP statistical software version 14 (SAS Institute, Cary, NC, USA). All reported *P* values are two sided, with *P*<0.05 taken as statistically significant. Receiver operating characteristics (ROC) analysis was performed to calculate the area under the curve (AUC) and to determine the best threshold for marker genes.

## Results

### Methylation analysis and marker selection using FNA samples

At first, we examined the genome-wide DNA methylation status of 37 FNA pancreatic cancer tissues with *KRAS* mutations using an Illumina Infinium HM450 Beadchip. The clinical features of the 37 pancreatic cancer cases are summarized in Table 1. Of 485,577 Ilumina Infinium probes, we identified 5,575 probes that were highly methylated in pancreatic cancers but not methylated in histologically normal pancreatic tissues (n = 3). After a comparison with the frequency of methylation in other cancer types, 1,933 probes were selected that were frequently methylated in pancreatic cancers. Since leukocytes in blood are the biggest source of cfDNA, we excluded probes that were methylated in normal whole blood samples (n = 95; GSE41169; see Materials and Methods, Fig 1A). After a comparison with DNA methylation data from normal tissues and blood, 420 genes were selected, that were substantially more highly methylated in cancerous compared with normal tissues.

**Table 1:** Clinical background of fine-needle aspiration (FNA) samples.

	Study set (n = 37)	Validation set (n = 46)	*P* value
Age, median (range)	66 (35–80)	65 (34–83)	0.97
Female (%)	17 (45.9)	17 (36.2)	0.50
Male (%)	20 (54.1)	29 (61.7)	
Stage (%)			
1 and 2	0 (0)	4 (9.3)	
3	11 (29.7)	11 (25.6)	0.57
4	25 (67.6)	30 (69.8)	
Unknown	1 (2.7)	1 (2.3)	
Location (%)			
Head	10 (27.0)	15 (34.9)	
Body-Tail	24 (64.9)	31 (64.9)	0.70
Unknown	2 (5.4)	0 (0)	
Tumor size (mm), median (range)	36.9 (20–63)	39.0 (22–90)	0.50
KRAS (%)			
Wild type	0 (0)	3 (6.5)	0.11
Mutation	37 (100)	43 (93.5)	

We next compared the DNA methylation status of the 420 genes in another nine common cancers (S2 Table, LUAD, LUSC, CRC, BRCA, BLCA, KICR, LAML, STAD, and SKCM) to pick up genes that were highly likely to diagnose pancreatic cancer but not other cancer types. We classified the 420 genes into three groups: 1) genes methylated in four cancer types, including pancreatic cancers; 2) genes methylated in two or three cancer types, including pancreatic cancers; and 3) genes only methylated in pancreatic cancers. Of the three criteria, we identified eight genes that were the most highly/frequently methylated in pancreatic cancers (*PCDH10*, *SPSB4*, *HOXA1*, *ADAMTS2*, *BMP3*, *C13orf18*, *CCND2*, and *SEMA5A*). Due to technical difficulties (e.g., extensively high CpG densities) in designing DNA methylation assays for eight genes close to differently methylated probes, DNA methylation assays were designed for only five genes, namely: ADAM metallopeptidase with thrombospondin type 1 motif 2 (*ADAMTS2*), homeobox A1 (*HOXA1*), protocadherin 10 (*PCDH10*), semaphorin 5A (*SEMA5A*), and SPRY domain-containing SOCS box protein 4 (*SPSB4*; Fig 1B). The promoter region of these genes showed dense CpG islands making it difficult to set up primers for pyrosequencing analysis, so some of the primers were designed for quantitative methylation specific PCR (qMSP).

We validated the five identified candidate genes by bisulfite pyrosequencing and/or qMSP analyses in FNA samples (n = 22 from the study set) as well as normal pancreatic tissues (n = 5) and chronic pancreatitis tissues (n = 6). The five genes were frequently found to be methylated in the 22 FNA samples. Further, these five genes were specifically methylated in a set of pancreatic cancers in comparison to five normal pancreatic tissues and six chronic pancreatitis tissues, although the mean DNA methylation levels of all five genes were not significantly different between normal and cancerous tissues (*P* = 0.051, 0.006, 0.001, 0.253, and 0.0871, for *ADAMTS2*, *HOXA1*, *PCDH10*, *SEMA5A*, and *SPSB4*, respectively). We determined the threshold in order to distinguish positive/negative DNA methylation in each gene by qMSP (*ADAMTS2*, *PCDH10*, *SEMA5A*, and *SPSB4*) or pyrosequencing analysis (*HOXA1*) using receiver operating characteristic curve (ROC) analysis. ROC analysis was performed between normal and pancreatic cancer tissues. Cut-off values of DNA methylation for *ADAMTS2*, *PCDH10*, *SEMA5A*, *SPSB4*, and *HOXA1* genes were 0.90, 0.70, 0.24, 0.20, and 17.64, respectively (S1A Fig).

### Validation study of markers in another cohort and TCGA data set

Next, we endeavored to establish diagnostic criteria for pancreatic cancers using the five marker genes. Notably, *KRAS* mutations are known to be the most frequently occurring mutations in pancreatic cancers [24, 25]. Therefore, we also examined whether the *KRAS* mutation status should be included in a diagnostic marker set in the current study.

DNA methylation analysis of five marker genes in a new set of 46 FNA pancreatic cancers and six benign pancreatic diseases (Table 1) revealed DNA methylation in at least one marker gene in all pancreatic cancer tissues, while benign pancreatic disease tissues did not show DNA methylation in the five marker genes (S1B Fig). *KRAS* mutations were found in 43 of 46 (93%) FNA samples. Notably, at least one of the marker genes was methylated in the three *KRAS* wild-type samples (S1B Fig).

Next, we examined the power of a combination of five marker genes and *KRAS* mutations in a larger sample size of pancreatic cancers using a public database. DNA methylation data that were analyzed by Illumina Infinium HM450 in 137 pancreatic ductal adenocarcinoma samples and 10 normal samples were obtained from a TCGA data set [26]. Most of the pancreatic cancer samples were stage II (n = 118, 86%; S3 Table). The DNA methylation status of multiple probes designed for CpG promoter regions (transcription start site ± 2kb) of five marker genes in the Illumina Infinium HM450 were analyzed for which regions we also designed qMSP and pyrosequencing analyses in FNA samples.

A total of 132 cases out of 137 pancreatic cancers showed DNA methylation in more than one marker gene (Fig 1C). The mean number of DNA methylated genes among the five marker genes per case was 3.5 ± 1.4. By contrast, only one sample showed DNA methylation in the five marker genes in 10 normal samples. For the TCGA data set, if we diagnosed pancreatic cancer when DNA methylation was positive in at least one marker gene in samples, the sensitivity and specificity of the five marker genes were 96% and 90%, respectively. Among the 118 stage II cancers in the TCGA database, the sensitivity and specificity of these markers were 98% and 90%, respectively, suggesting a potential diagnostic capability even at relatively early stages of pancreatic cancers.

We further examined *KRAS* mutations in the TCGA data set. Of 137 pancreatic cancers, 36, 59, and 42 were *KRAS* wild type, *KRAS* mutations, and of an unknown mutation status, respectively. Two pancreatic cancers with *KRAS* wild type (5.6%) did not show DNA methylation in the five marker genes, while all cancers with *KRAS* mutations had DNA methylation in at least one marker. When DNA methylation markers were combined with the *KRAS* mutation status (diagnosed as pancreatic cancer when *KRAS* mutation positive or/and DNA methylation positive in at least one marker gene), the sensitivity was further improved to 98%.

### Establishment of MBD-ddPCR assay

**Fig 2:**
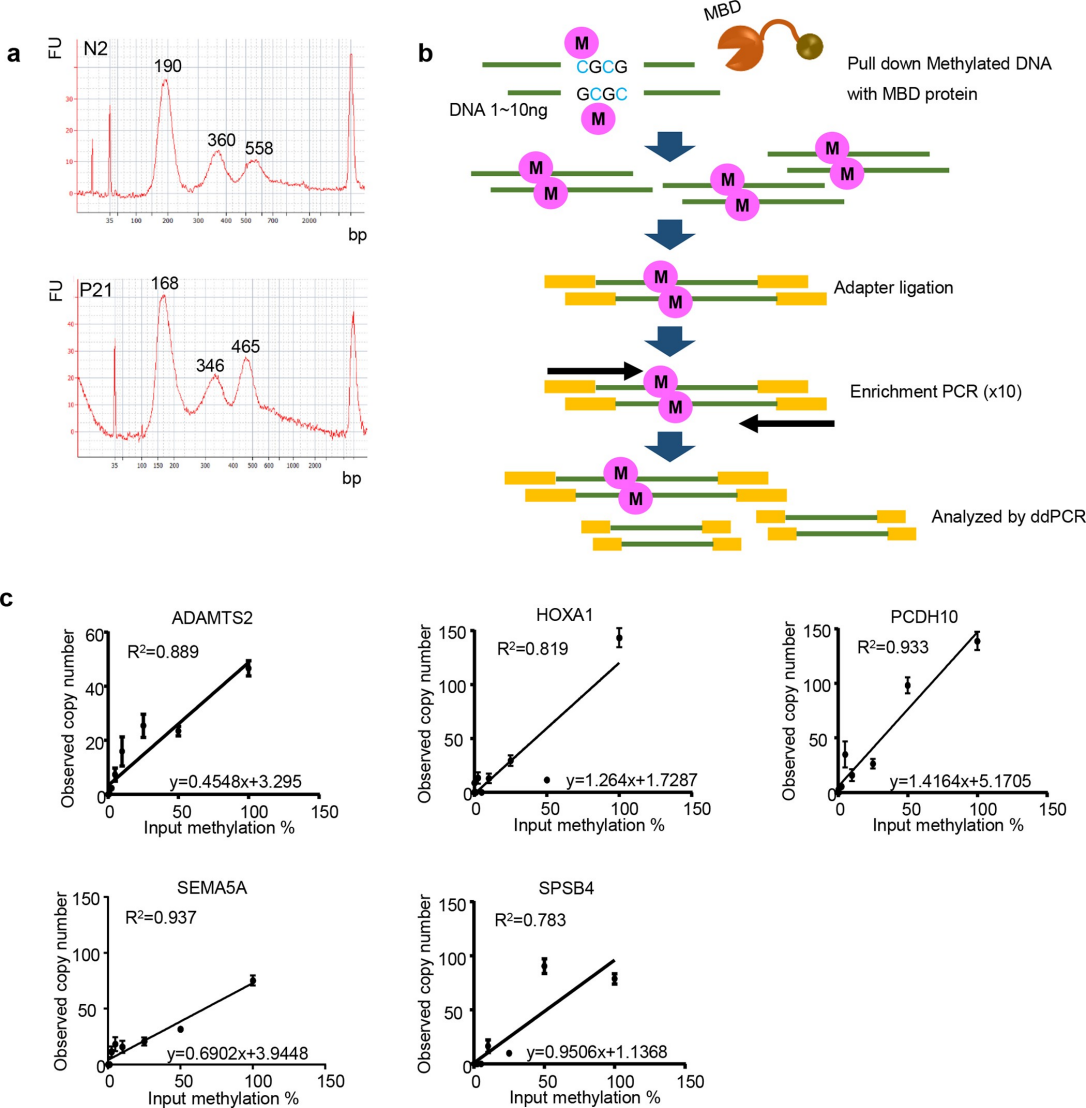
Length of cfDNA and outline of MBD–ddPCR. (a) Size distribution of cfDNA length analyzed by Bioanalyzer. Upper panel, cfDNA from a normal healthy volunteer; lower panel, cfDNA from a patient with pancreatic cancer. (b) Outline of methyl-CpG binding domain (MBD) by droplet digital PCR (MBD–ddPCR) method. A methylated DNA fragment was precipitated with MBD protein. The methylated DNA fragment was ligated to adaptors, amplified with an adaptor sequence, and then analyzed by ddPCR. (c) MBD–ddPCR in control samples. At first, copy numbers of 100% methylated and 0% methylated DNA were analyzed by ddPCR. Methylated DNA was mixed with unmethylated DNA in different ratios (0, 0.1, 0.5, 1, 2.5, 5, 10, 25, 50, 100% methylation) in a total amount that corresponds to 1,000 copies of each single gene quantified by ddPCR. DNA methylation ratio of input are indicated on the x-axis. Observed methylated copy numbers are indicated on the y-axis.

Cell-free DNA contains small fragments of around 200 bp as well as larger fragments in agreement with a previous report [27] (Fig 2A). Since the amount of cfDNA was relatively low in serum samples (S4 Table), we could not reproducibly detect DNA methylation in five marker genes in cfDNA by pyrosequencing analysis or qMSP, which are bisulfite-based DNA methylation assays that sometimes suffer from DNA loss/degradation during bisulfite treatment and subsequent processes (S2A Fig). Therefore, we set up a new method based on MBD [28] immunoprecipitation coupled with droplet digital PCR (i.e., MBD–ddPCR; Fig 2B). This method is highly sensitive since DNA fragments captured by MBD are impartially amplified by adapter primers before ddPCR in order to apply a very limited amount of cfDNA sample. Methylated DNA was mixed with unmethylated DNA in different ratios (0, 0.1, 0.5, 1, 2.5, 5, 10, 25, 50, 100% methylation) in a total amount that corresponded to 1,000 copies per each single gene and detection capability of DNA methylation examined (Fig 2C). DNA methylation levels (mixture of unmethylated and methylated DNA) and DNA methylation levels examined by MBD–ddPCR were highly correlated with each other in all five marker genes (*ADAMTS2*, *R*^*2*^ = 0.889; *HOXA1*, *R*^*2*^ = 0.819; *PCDH10*, *R*^*2*^ = 0.933; *SEMA5A*, *R*^*2*^ = 0.937; *SPSB4*, *R*^*2*^ = 0.783). From the control samples, the following formulae were acquired, *ADAMTS2*, y = 0.4548x+3.295; *HOXA1*, y = 1.2164x+1.7287; *PCDH10*, y = 1.4164x+5.1705; *SEMA5A*, y = 0.6902x+3.9448; *SPSB4*, y = 0.9506x+1.1368 (x and y indicate methylation percentage of input DNA and observed copy number, respectively; Fig 2C). Further, we found that if more than 100 copies of methylated DNA fragments corresponding to each gene were included in the samples, we could successfully detect these by MBD–ddPCR assay (S5 Table).

### MBD–ddPCR analysis in pancreatic cancers

**Fig 3:**
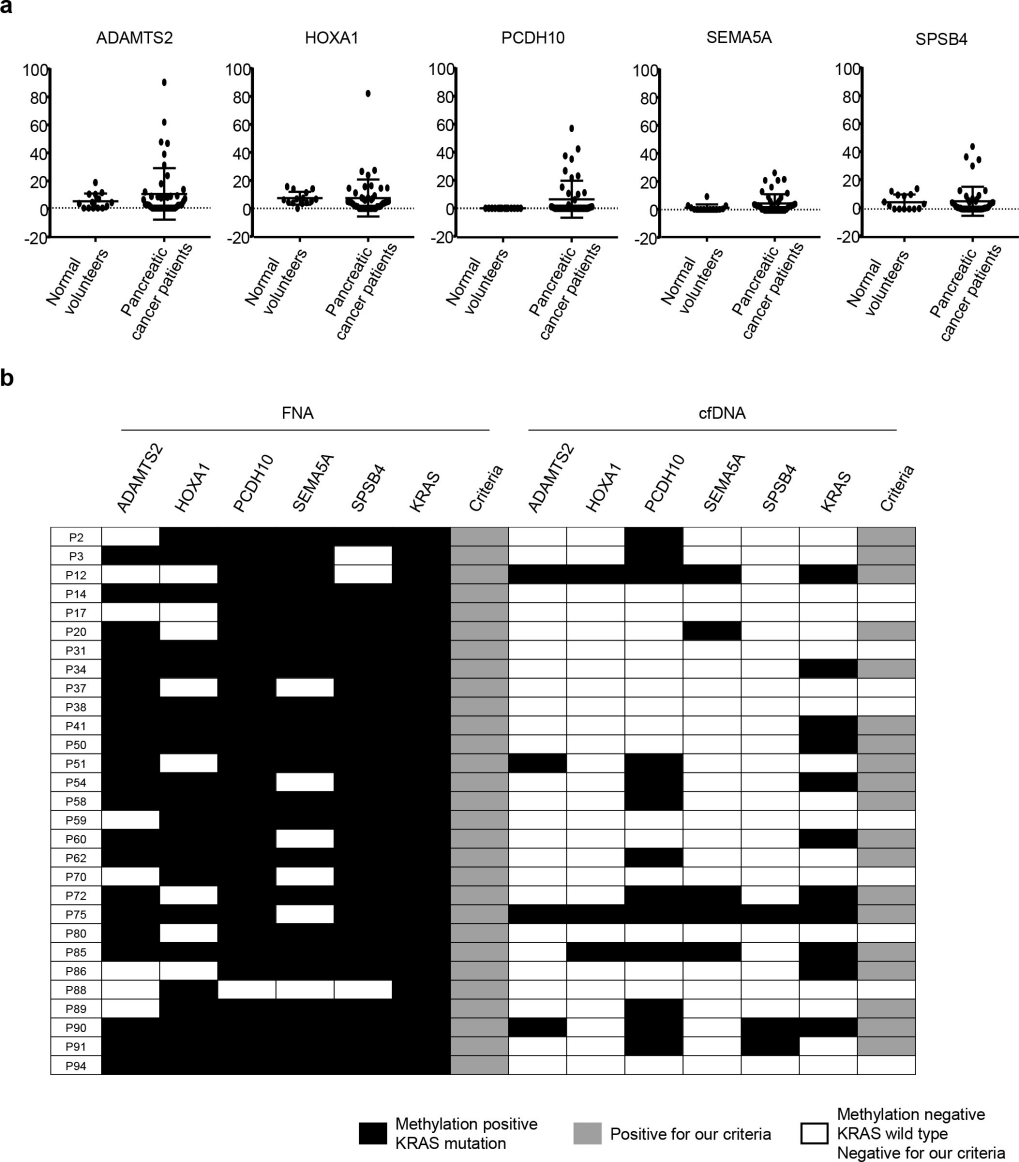
Methylation status of the five marker genes in cfDNA analyzed by MBD–ddPCR. (a) Methylation level of five markers in cfDNA from normal health controls (n = 14) and patients with pancreatic cancer (n = 47). DNA methylation levels of each gene are indicated on the y-axis. Horizontal lines represent the mean and error bars represent standard deviation. (b) Methylation and *KRAS* mutation status of paired samples of fine needle aspiration (FNA) and cfDNA (n = 29). The methylation status of FNA samples was analyzed by pyrosequencing or qMSP. The methylation status of cfDNA was analyzed by MBD–ddPCR. Black boxes, methylation or *KRAS* mutation positive; gray boxes, positive in our criteria (at least one methylation marker or *KRAS* mutation positive); white boxes, methylation or *KRAS* mutation negative or negative in our criteria.

The DNA methylation status of five marker genes in serum samples from patients with pancreatic cancer (n = 47) and normal volunteers (n = 14) was analyzed by MBD–ddPCR and the DNA methylation level calculated using the formula mentioned above. A certain population of pancreatic cancers showed substantially higher methylation levels, although mean methylation levels of cfDNA in five marker genes of normal volunteers and cancer patients were not significantly different (Fig 3A; *P* = 0.31, 0.98, 0.07, 0.09, and 0.83, in *ADAMTS2*, *HOXA1*, *PCDH10*, *SEMA5A*, and *SPSB4*, respectively). Twenty-three (49%) cfDNA samples were methylation positive in at least one of the five marker genes (sensitivity and specificity were 49% and 86%, respectively; S2B Fig). Among the five marker genes, *SPSB4* showed a relatively low potential compared to the other four markers (Fig 2C, S2B Fig). However, in tissue samples, *SPSB4* was still a potential marker with highest AUC among five markers (AUC = 0.9938, *P*<0.0001). In addition, there was a tissue sample which showed methylation positive only in *SPSB4* (S1B Fig). Therefore, we included *SPSB4* as diagnosis marker of cfDNA to improve the sensitivity.

Further, we analyzed *KRAS* mutation status by ddPCR and found that 23 (49%) samples showed *KRAS* mutations in cfDNA, while *KRAS* mutations was detected in all corresponding FNA samples. When the DNA methylation and *KRAS* mutation status were combined as diagnostic markers in cfDNA, the sensitivity and the specificity improved to 68% and 86%, respectively. DNA methylation and *KRAS* mutation status were further compared between paired FNA and cfDNA samples (n = 29). While all the FNA samples were DNA methylation positive and *KRAS* mutation positive, 19 (66%) cfDNA samples were either DNA methylation positive or *KRAS* mutation positive (Fig 3B). Taken together, we defined the diagnostic criterion for pancreatic cancer as *KRAS* mutation positive, and/or DNA methylation positive in at least one marker gene in cfDNA samples.

Regarding clinical background, we found that pancreatic cancers that were DNA methylation positive and/or *KRAS* mutation positive in cfDNA were significantly associated with larger sized tumors (36.2 ± 9.0 vs. 45.7 ± 12.6, *P* = 0.04) and a higher frequency of liver metastasis (20% vs. 53%, *P* = 0.02), indicating more methylated DNA in circulating blood from advanced pancreatic cancers (S6 Table).

### Genome-wide comparison of methylation status between FNA and cfDNA

**Fig 4:**
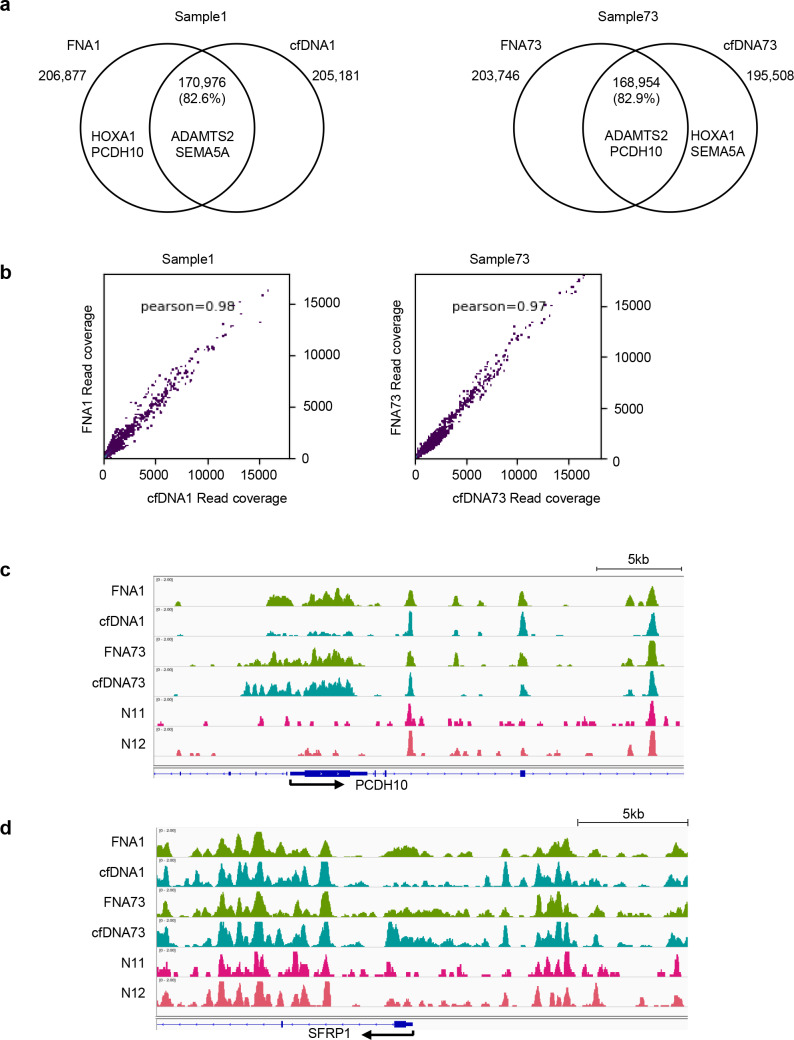
MBD-seq analysis of FNA and paired cfDNA samples. (a) Venn diagram showing overlap of methylation peaks from fine-needle aspiration (FNA) of pancreatic cancer and cfDNA samples from the same patient. The gene names in the circle indicate that their methylation peaks were identified in each sample. (b) Genome-wide correlation between FNA and cfDNA profiles. Pearson's correlation coefficient, r, is shown in the graph. (c, d) Visualization of MBD–seq data in Integrative Genome Viewer (IGV). (c) The promoter region of *PCDH10*, (d) the promoter region of *SFRP1*. N11 and N12 are cfDNA samples from normal healthy controls.

Heterogeneity in DNA methylation status may exist between primary tumors and cfDNA. We compared the genome-wide DNA methylation status between FNA and cfDNA samples from the same patients. MBD-sequencing (seq) was performed in paired samples of FNA and cfDNA from two patients. Using HOMER software, we identified DNA methylation peaks in each sample (Fig 4A).

The majority of DNA methylation peaks overlapped in FNA and cfDNA samples (82.6% and 82.9% in patients #1 and #73, respectively). DNA methylation levels of each gene in FNA and cfDNA samples were highly correlated with each other (r = 0.98 and 0.97 in samples 1 and 73, respectively; Fig 4B). Interestingly, among the five marker genes, *ADAMTS2* and *SEMA5A* were methylated in both FNA and cfDNA samples, while *HOXA1* and *PCDH10* were methylated in only an FNA sample in patient #1. *ADAMTS2* and *PCDH10* were methylated in both FNA and cfDNA samples, while *HOXA1* and *SEMA5A* were methylated in only a cfDNA sample in patient #73. DNA methylation peaks by MBD-seq analysis are shown in Fig 4C and 4D. The promoter region of *SFRP1* is often methylated in cfDNA from pancreatic cancer patients [29], so we also focused on this region (Fig 4D). While no peak calls in *PCDH10* and *SFRP1* were detected in cfDNA from normal healthy volunteers (N11 and N12), substantial peaks were observed in either or both cancerous and cfDNA samples, indicating that methylation of this region is cancer specific in these samples.

## Discussion

Cell-free DNA is released from tumors into circulating blood and contains tumor specific mutations, methylation sites, and miRNAs. Since it reflects information that is characteristic of primary tumors, cfDNA in plasma or serum could be used as a “liquid biopsy” that may be useful for the diagnosis and monitoring of cancer, avoiding sequential tissue biopsies [3]. In the current study, we identified five characteristic methylation marker genes from pancreatic cancers that can be combined with our highly sensitive DNA methylation detection method for cfDNA from cancer patients. Detecting characteristic mutations at an early stage in the development of pancreatic cancer may be ideal for diagnosis and effective therapy. However, this may be challenging because of the limited number of DNA copies with certain specific and frequently mutated genes in cfDNA from cancer patients [30]. In the cfDNA analysis of this study, all paired FNA samples were derived from advanced pancreatic cancers with *KRAS* mutations; the detection rate of *KRAS* mutations in cfDNA by ddPCR was not very high (49%). In agreement with our study, a recent investigation showed that 43% of rectal cancer patients were *KRAS* mutation positive for paired cfDNA from those with *KRAS* mutations [31]. These data suggest that the detection of genetic mutations alone appears not to be sensitive enough for a cancer diagnosis using cfDNA. In most of the recent cfDNA study, cfDNA was obtained from plasma samples since that in serum contains more background DNA released from blood cells during the clotting process than samples from plasma [32]. Since our cfDNA samples were collected from serum, contaminating DNA from blood cells may have lowered the sensitivity and specificity of our cfDNA sample.

CpG methylation induces tighter wrapping of DNA around the histone core, accompanied by a topology change, and confers nucleosome stability [33]. Unmethylated CpG islands near transcription start sites became enriched in nucleosomes upon methylation [34]. Our study showed that a DNA fragment length of 150–200 bp was most prevalent in cfDNA (Fig 2A), which may reflect nucleosome core fragment sizes. Therefore, methylated DNA is more stable in serum than unmethylated DNA. The detection of DNA methylation may also be easier than unmethylated DNA fragments in liquid biopsy.

Since each cell type has a unique DNA methylation pattern, this has the potential to identify the tissue of origin. Shen et al. showed that differentially methylated regions from cfDNA could distinguish between tumor types. Their classifier, based on methylation profile, discriminated between cancerous and normal tissues, even at an early stage of disease [35]. In the current study, we identified five characteristic marker genes: *ADMATS2* is a metalloproteinase that is known to have anti-tumor effects [36]. Dysregulation of *HOX* genes is associated with cancer and are often methylated in many cancer types, as well as in pancreatic cancer [37]. *PCDH10*, a member of the protocadherin family, is frequently mutated in pancreatic cancer [38]; it is known as a tumor suppressor and is often silenced by DNA methylation in many types of cancer [39] [40]. Semaphorin signaling seems to play an important role in the development of pancreatic cancer, with a high frequency of mutations in *SEMA3A* and *SEMA3B* [41]. And *SPSB4* is a SPRY domain- and SOCS box-containing protein that is known as an E3 ubiquitin ligase [42]. Although the precise roles of the five marker genes in pancreatic tumorigenesis are not completely understood, our data highlighted the promising diagnostic potential of these genes as markers of this disease.

Another important issue is consistency in DNA methylation status between primary tissue samples and cfDNA. Our genome-wide analysis of DNA methylation in the same patients using sensitive MBD-seq revealed that the majority of the DNA methylation profiles between the two were common (more than 82%), though several characteristic DNA methylation profiles were also observed in each. Indeed, multiple marker genes among the five marker genes were commonly methylated in both FNA samples and cfDNA. Although pancreatic cancers generally show tissue heterogeneity, as was found with DNA, our data indicated that sensitive methods based on multiple DNA methylation markers in cfDNA may become a promising approach in the diagnosis of pancreatic cancer.

A recent clinical trial, the Circulating Cell-free Genome Atlas (CCGA) study, analyzed the genome-wide DNA methylation status to diagnose cancer and identify its tissue of origin (NCT02889978). From an analysis using whole-genome bisulfite sequencing (WGBS) coupled with a targeted sequence, the overall sensitivity was found to be 76% in 13 cancer types (https://grail.com). Our analysis using five DNA methylation markers plus the *KRAS* mutation status, analyzed by the novel method of cfDNA samples was slightly less sensitive (68%) than the CCGA study. However, the amount of DNA required in our study was relatively low (i.e., 10 ng). In addition, our method can quickly yield results (i.e., 2–3 days). Further analysis, such as using the CCGA, will provide us with other possible DNA methylation markers for each type of cancer in order to use this method in future cancer screenings.

In summary, we have demonstrated a potent approach for the diagnosis of pancreatic cancer using cfDNA methylation status. Although further optimization may be required for practical clinical use, the combination of DNA methylation markers with highly sensitive MBD–ddPCR may provide a non-invasive effective diagnostic strategy for identifying devastating pancreatic cancers.

## Supporting information

S1 Table Sequence of primers. (DOCX)

S2 Table List of top 20 genes in each class to the selection of candidate markers from 420 genes. (DOCX)

S3 Table Clinical feature of the cancer genome atlas (TCGA) samples. (DOCX)

S4 Table cfDNA concentration. (DOCX)

S5 Table Copy number after methyl-CpG binding domain (MBD) enrichment and amplification in control samples analyzed by MBD- droplet digital PCR. (DOCX)

S6 Table Clinical background of marker positive and negative in cfDNA. (DOCX)

S1 Fig Methylation status in FNA samples. (a) ROC curve of marker gene. ROC curves to set the thresholds of the markers using study set of FNA samples (n = 22). Cut off values are indicated in the graph, (b) Methylation and KRAS mutation status of validation set of fine needle aspiration (FNA) samples (n = 46) and benign pancreatic disease (n = 6). Black boxes, methylation or KRAS mutation positive; white boxes, methylation or KRAS mutation negative.
(TIF)

S2 Fig MBD-ddPCR analysis in cfDNA. (a) qMSP analysis of low input DNA. 10,000, 1,000, 500, and 100 copy number of DNA (same DNAwas used as S2A Fig) was used for bisulfite treatment. Eluted DNA (total 20pl) were then analyzed by qMSP (1 pl of DNAwas used for each well). Copy number for bisulfite are indicated on the x-axis. Cycle number were indicated on the y-axis. nd, not detected, (b) Methylation and KRAS mutation status of cfDNA (cancer patient, normal volunteer, n = 47 and 14, respectively) analyzed by MBD-ddPCR. Black boxes, methylation or KRAS mutation positive; white boxes, methylation or KRAS mutation negative.
(TIF)
