# Supplementary material for: A novel sensitive detection method for DNA methylation in circulating free DNA of pancreatic cancer
Source: PLoS One. 2020 Jun 10;15(6):e0233782. doi: 10.1371/journal.pone.0233782 (PMC7286528; doi:10.1371/journal.pone.0233782)
Supplement: S1 Table — (DOCX) [file pone.0233782.s001.docx]

Supplementary Table 1. Sequence of primers

**Primers for Pyrosequencing analysis**

| Gene name | Sequence | Condition (°C) |
| --- | --- | --- |
| HOXA1-F | TTTTATGGAGGAAGTGAGAAAGT | 52.5 |
| HOXA1-R | GGGACACCGCTGATCGTTTATCCAAAAAAAAATTCATTCTTACA |  |
| HOXA1-Sequencing | AAGTGAGAAAGTTGGTATAG |  |
| Universal primer | Biotin-GGGACACCGCTGATCGTTTA |  |

**Primers for quantitative methylation specific PCR**

| Gene name | Sequence | Condition (°C) |
| --- | --- | --- |
| ADAMTS2-F | TTTCGGGGTGTTTTTGTTTC | 60 |
| ADAMTS2-R | TAACCCGACGCATCTTAACG |  |
| ADAMTS2-probe | FAM/CG AGT TCG G/ZEN/T ATC GCG GCG AC/3IABkFQ |  |
| PCDH10-F | TTGGGGATTGGGAATTTTTC | 60 |
| PCDH10-R | ACAACCGAACGAAACGAAAC |  |
| SEMA5A-F | GGTTTCGTAGTTTTAGGTTAGTCGC | 62 |
| SEMA5A-R | GAAATAAACGACACTAACCGAACCG |  |
| SPSB4-F | GGCGTTTGAGCGTTAATTTC | 60 |
| SPSB4-R | CGCAAATACCGACTCTCTCC |  |
| LINE1-F | TTTTAAAGTTGTTAGATAGGGATATTTAAGTTTGTA | 56 |
| LINE1-R | AAAAACCTACCTACCTCTATAAACTCCACC |  |

**Primers for MBD-droplet digital PCR**

| Gene name | Sequence | Condition (°C) |
| --- | --- | --- |
| ADAMTS2-F | GCTCCTCGGAGGTTGTGC | 55 |
| ADAMTS2-R | TCGCAGCGAAGCAGAGAC |  |
| ADAMTS2-probe | VIC-AGGCGGCGGCAGA |  |
| PCDH10-F | GATTGGTTGGCAGAATGAGG | 60 |
| PCDH10-R | GGCTGGCAGTTTCTGAGC |  |
| PCDH10-probe | FAM-AAAACGGAGAAGCCGAGC |  |
| SEMA5A-F | GGCCAGTGTCGCTCATCC | 55 |
| SEMA5A-R | AGGTGGCAAAGTTGGGTGT |  |
| SEMA5A-probe | FAM-AAGCCAGCTCCGCG |  |
| SPSB4-F | CCAGGATCGCTTCAGTAAGG | 60 |
| SPSB4-R | AGGATGGCCTGGAGTTAGGA |  |
| SPSB4-probe | VIC-GCGCCAACTTCGCCAA |  |
| HOXA1-F | CCCATGGAGGAAGTGAGAAA | 55 |
| HOXA1-R | GGGGTATTCCAGGAAGGAGT |  |
| HOXA1-probe | FAM-GCACAGTCACGCCGG |  |

**Primers for enrichment of methylated DNA**

Name Sequence

| Adaptor | ACACTCTTTCCCTACACGACGCTCTTCCGATC*T |
| --- | --- |
|  | /5Phos/GATCGGAAGAGCACACGTCTGAACTCCAGTCACCTACCAGGATCTCGTATGCCGTCTTCTGCTTG |
| Amplification-F | CAAGCAGAAGACGGCATACGAGAT |
| Amplification-R | ACACTCTTTCCCTACACGAC |

**Control oligos for MBD**

Name**/** Sequence

| YLR255C-M-sense/ GAGGAAGAGAGAGAGAGAGAA‍AGGGCACAC^m^GAAATTCAGGATAC^m^GGC^m^GGAGGAGTTATCTTTATTTTATAC^m^GGTCTTGCCTTGTAAGGCCCTACTCAAGC^m^GGGAACAAGAAAAACAGTTG  YLR255C-M-antisense/  CAACTGTTTTTCTTGTTCCC^m^GCTTGAGTAGGGCCTTACAAGGCAAGACC^m^GTATAAAATAAAGATAACTCCTCC^m^GCC^m^GTATCCTGAATTTC^m^GTGTGCCCTTTCTCTCTCTCTCTCTTCCTC  YPR071W-UM-sense/  GCGATCGTTTCTATTTGGGGGTTTGCTGTGTGGATGGAAAGAGGATATAGACATAAGATTAATCTACTGCCTCCAAGATGTACGAAGATAAGATGCTCTCGCTGCAATACAAGAATAAGA  YPR071W-UM-antisense/  TCTTATTCTTGTATTGCAGCGAGAGCATCTTATCTTCGTACATCTTGGAGGCAGTAGATTAATCTTATGTCTATATCCTCTTTCCATCCACACAGCAAACCCCCAAATAGAAACGATCGC |
| --- |

MBD, methyl-CpG binding domain; C^m^, 5-methyl cytosine; M, methylated oligo; UM, unmethylated oligo

* indicates phosphorothioate
