# Supplementary material for: A novel sensitive detection method for DNA methylation in circulating free DNA of pancreatic cancer
Source: PLoS One. 2020 Jun 10;15(6):e0233782. doi: 10.1371/journal.pone.0233782 (PMC7286528; doi:10.1371/journal.pone.0233782)
Supplement: S2 Table — (DOCX) [file pone.0233782.s002.docx]

Supplementary Table 2. List of top 20 genes in each class to the selection of candidate markers from 420 genes.

|  | Gene symbol | Frequency of methylation in PCa* (%) | Probe number in CpG islands | | | Methylated probes in CpG island in PCa (%) | | | Methylation positive close to TSS*  (< ±2000bp) in normal blood | | | Difficult to make suitable primers | | Selected as a candidate gene |
| --- | --- | --- | --- | --- | --- | --- | --- | --- | --- | --- | --- | --- | --- | --- |
| Methylated in more than 4 different types of cancers | | | | |  | |  | | |  |  |  |  |  |
|  | GFRA1 | 70.9 | 33 | | | 33.3 | | | + | | |  | |  |
|  | KCNA1 | 88.9 | 18 | | | 50.0 | | | + | | |  | |  |
|  | PCDH10 | 87.2 | 19 | | | 47.4 | | |  | | |  | | + |
|  | OLIG1 | 76.8 | 16 | | | 50.0 | | | + | | | + | |  |
|  | GATA5 | 58.0 | 30 | | | 26.7 | | | + | | |  | |  |
|  | ST6GALNAC5 | 82.8 | 12 | | | 66.7 | | |  | | | + | |  |
|  | SFMBT2 | 65.7 | 28 | | | 25.0 | | | + | | |  | |  |
|  | ELMO1 | 83.2 | 12 | | | 58.3 | | |  | | |  | |  |
|  | RIMS2 | 76.9 | 16 | | | 43.8 | | |  | | |  | |  |
|  | SPSB4 | 78.1 | 21 | | | 28.6 | | |  | | |  | | + |
|  | NTRK3 | 80.2 | 10 | | | 60.0 | | |  | | |  | |  |
|  | RXFP3 | 73.9 | 18 | | | 33.3 | | |  | | |  | |  |
|  | ZNF542 | 81.5 | 10 | | | 60.0 | | |  | | |  | |  |
|  | VAT1L | 79.7 | 7 | | | 85.7 | | |  | | |  | |  |
|  | TMEM132C | 75.7 | 20 | | | 30.0 | | |  | | |  | |  |
|  | FOXI3 | 64.5 | 11 | | | 54.5 | | |  | | |  | |  |
|  | ATP10A | 77.4 | 27 | | | 22.2 | | |  | | | + | |  |
|  | CUGBP2 | 76.7 | 16 | | | 37.5 | | |  | | |  | |  |
|  | RASGRF1 | 66.4 | 23 | | | 21.7 | | |  | | |  | |  |
|  | IRX4 | 77.9 | 25 | | | 20.0 | | |  | | |  | |  |
| Methylated in 3-2 types of cancers | | | |  | | | |  | | |  | |  |  |
|  | HOXA1 | 86.2 | 14 | | | 64.3 | | |  | | |  | | + |
|  | PHF21B | 75.2 | 28 | | | 28.6 | | |  | | |  | |  |
|  | ADAMTS2 | 78.8 | 39 | | | 20.5 | | |  | | |  | | + |
|  | GDNF | 77.5 | 29 | | | 27.6 | | | + | | |  | |  |
|  | ST8SIA4 | 65.2 | 10 | | | 80.0 | | |  | | |  | |  |
|  | SH3GL3 | 73.9 | 10 | | | 70.0 | | |  | | |  | |  |
|  | CNIH3 | 70.3 | 13 | | | 53.8 | | |  | | |  | |  |
|  | BMP3 | 74.7 | 9 | | | 66.7 | | |  | | |  | | + |
|  | GUCY1A2 | 67.9 | 13 | | | 46.2 | | |  | | |  | |  |
|  | AMPH | 88.7 | 7 | | | 85.7 | | |  | | |  | |  |
|  | C13orf18 | 67.6 | 7 | | | 71.4 | | |  | | |  | | + |
|  | NEUROG1 | 65.6 | 17 | | | 29.4 | | | + | | |  | |  |
|  | BNIP3 | 72.1 | 20 | | | 25.0 | | | + | | | + | |  |
|  | RET | 72.1 | 16 | | | 31.3 | | |  | | |  | |  |
|  | HMX3 | 53.6 | 21 | | | 23.8 | | |  | | |  | |  |
|  | CACNB2 | 95.0 | 9 | | | 55.6 | | |  | | |  | |  |
|  | IGFBP3 | 66.8 | 11 | | | 45.5 | | |  | | |  | |  |
|  | COL23A1 | 69.9 | 23 | | | 21.7 | | |  | | |  | |  |
|  | FADS2 | 58.9 | 11 | | | 45.5 | | |  | | |  | |  |
|  | TFAP2C | 68.9 | 13 | | | 30.8 | | |  | | | + | |  |
| Methylated in only pancreatic cancer | | | |  | | | |  | | |  | |  |  |
|  | TBX3 | 64.5 | 21 | | | 23.8 | | |  | | | + | |  |
|  | TSPAN2 | 64.2 | 8 | | | 25.0 | | | + | | | + | |  |
|  | CCND2 | 61.8 | 35 | | | 34.3 | | |  | | |  | | + |
|  | HIST1H4E | 52.7 | 7 | | | 14.3 | | |  | | |  | |  |
|  | FSD1 | 50.0 | 10 | | | 20.0 | | | + | | |  | |  |
|  | SEMA5A | 47.3 | 10 | | | 10.0 | | |  | | |  | | + |
|  | ZNF695 | 40.5 | 6 | | | 16.7 | | | + | | |  | |  |

*, PCa, pancreatic cancer; TSS, transcriptional start site
