# Supplementary material for: A novel sensitive detection method for DNA methylation in circulating free DNA of pancreatic cancer
Source: PLoS One. 2020 Jun 10;15(6):e0233782. doi: 10.1371/journal.pone.0233782 (PMC7286528; doi:10.1371/journal.pone.0233782)
Supplement: S3 Table — (DOCX) [file pone.0233782.s003.docx]

Supplementary Table 3. Clinical feature of the cancer genome atlas (TCGA) samples.

| Characteristics | Number (%) |  |
| --- | --- | --- |
| Age, median (range), y | 65.2 (35-88) |  |
|  |  |  |
| Female | 64 (46.7) |  |
| Male | 73 (53.3) |  |
|  |  |  |
| Stage |  |  |
| 1 | 11 (8.0) |  |
| 2 | 118 (86.2) |  |
| 3 | 4 (2.9) |  |
| 4 | 4 (2.9) |  |
| History of chronic pancreatitis | |  |
| Yes | 13 (9.4) |  |
| No | 94 (68.6) |  |
| Unknown | 30 (22.0) |  |
| History of diabetes | |  |
| Yes | 30 (21.9) |  |
| No | 82 (59.9) |  |
| Unknown | 25 (18.2) |  |
| Tumor size (mm), median (range) | 37.7 (15-120) |  |
| KRAS |  |  |
| Wild type | 36 (26.3) |  |
| Mutation | 59 (43.1) |  |
| No data | 42 (30.7) |  |
