# Supplementary material for: A novel sensitive detection method for DNA methylation in circulating free DNA of pancreatic cancer
Source: PLoS One. 2020 Jun 10;15(6):e0233782. doi: 10.1371/journal.pone.0233782 (PMC7286528; doi:10.1371/journal.pone.0233782)
Supplement: S4 Table — (DOCX) [file pone.0233782.s004.docx]

Supplementary Table 4. cfDNA concentration

|  | Normal volunteers (n=8) | Pancreatic cancer patients (n=47) | *P* value |
| --- | --- | --- | --- |
| Total DNA in 1ml serum (ng/ml) (mean ± sd) (range) | 72.0 ± 58.9  (5.6-146.8) | 102.6 ± 69.4  (19.9-286.8) | 0.21 |

sd, standard deviation
