# Supplementary material for: A novel sensitive detection method for DNA methylation in circulating free DNA of pancreatic cancer
Source: PLoS One. 2020 Jun 10;15(6):e0233782. doi: 10.1371/journal.pone.0233782 (PMC7286528; doi:10.1371/journal.pone.0233782)
Supplement: S5 Table — (DOCX) [file pone.0233782.s005.docx]

Supplementary Table 5. Copy number after methyl-CpG binding domain (MBD) enrichment and amplification in control samples analyzed by MBD- droplet digital PCR

Copy number

| Copy number of methylated DNA | Input methylation % | HOXA1 | ADAMTS2 | SEMA5A | PCDH10 | SPSB4 |
| --- | --- | --- | --- | --- | --- | --- |
| 1000 | 100 | 143.2 | 46.6 | 75.4 | 138.6 | 78.8 |
| 500 | 50 | 11.4 | 23.4 | 31.7 | 98.2 | 90.6 |
| 250 | 25 | 29.4 | 25.5 | 20.7 | 26.3 | 9.9 |
| 100 | 10 | 13.0 | 15.9 | 15.9 | 15.9 | 16.6 |
| 50 | 5 | 0.0 | 7.2 | 18.4 | 34.8 | 0.0 |
| 25 | 2.5 | 13.2 | 2.2 | 11.5 | 5.5 | 0.0 |
| 10 | 1 | 0.0 | 0.4 | 0.0 | 4.9 | 0.0 |
| 5 | 0.5 | 0.0 | 0.0 | 0.0 | 0.0 | 0.0 |
| 1 | 0.1 | 8.6 | 0.0 | 0.0 | 2.5 | 0.0 |
| 0 | 0 | 0.0 | 0.0 | 0.0 | 0.0 | 0.0 |

A total of 1,000 copies of DNA fragment was used as starting DNA amount for MBD.

The copy number of methylated DNA fragments was calculated by the copy number of DNA fragments in MBD enriched sample/ input sample.
