# Supplementary material for: A novel sensitive detection method for DNA methylation in circulating free DNA of pancreatic cancer
Source: PLoS One. 2020 Jun 10;15(6):e0233782. doi: 10.1371/journal.pone.0233782 (PMC7286528; doi:10.1371/journal.pone.0233782)
Supplement: S6 Table — (DOCX) [file pone.0233782.s006.docx]

Supplementary Table 6. Clinical background of marker positive and negative in cfDNA

|  | Negative (n=15) | Positive (n=32) | *P* value |
| --- | --- | --- | --- |
| Age (mean ± sd) | 63.2 ± 9.36 | 64.7 ± 11.85 | 0.60 |
| Stage (%) |  |  |  |
| 2 | 2 (13.3) | 0 (0) |  |
| 3~4 | 11 (73.4) | 30 (93.7) | 0.03 |
| Unknown | 2 (13.3) | 2 (11.1) |  |
| Tumor size (mm) (mean ± sd) | 36.2 ± 9.0 | 45.7 ± 12.6 | 0.04 |
| CA19-9 (U/ml) (mean ± sd) | 3841 ± 7363 | 8886 ± 22582 | 0.39 |
| CEA (ng/ml) (mean ± sd) | 5.7 ± 7.8 | 31.6 ± 99.8 | 0.34 |
| DUPAN2 (U/ml) (mean ± sd) | 1900 ± 3831 | 2856 ± 6099 | 0.67 |
| Serum DNA concentration (ng/1ml serum)  (mean ± sd) | 113.3 ± 60.0 | 97.5 ± 73.7 | 0.47 |
| Liver metastasis positive cases (%) | 3 (20.0) | 17 (53.1) | 0.02 |

sd, standard deviation; CA19-9, carbohydrate antigen 19-9; CEA, carcinoembryonic antigen; DUPAN2, detection of a pancreatic cancer-associated antigen
