# Supplementary figures and images for: A novel sensitive detection method for DNA methylation in circulating free DNA of pancreatic cancer
Source: PLoS One. 2020 Jun 10;15(6):e0233782. doi: 10.1371/journal.pone.0233782 (PMC7286528; doi:10.1371/journal.pone.0233782)

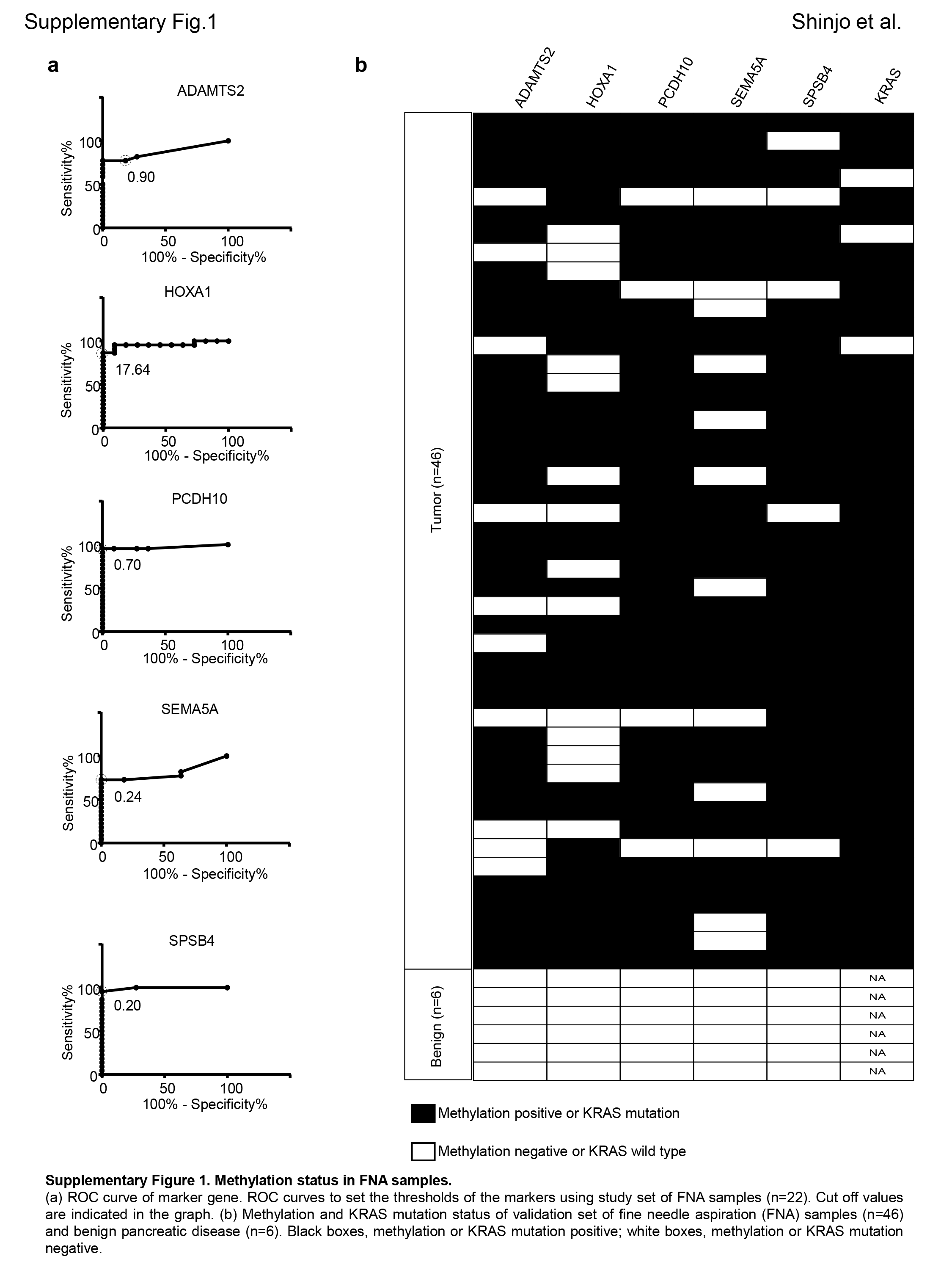

Supplement: S1 Fig — (a) ROC curve of marker gene. ROC curves to set the thresholds of the markers using study set of FNA samples (n = 22). Cut off values are indicated in the graph, (b) Methylation and KRAS mutation status of validation set of fine needle aspiration (FNA) samples (n = 46) and benign pancreatic disease (n = 6). Black boxes, methylation or KRAS mutation positive; white boxes, methylation or KRAS mutation negative. (TIF) [file pone.0233782.s007.tif]

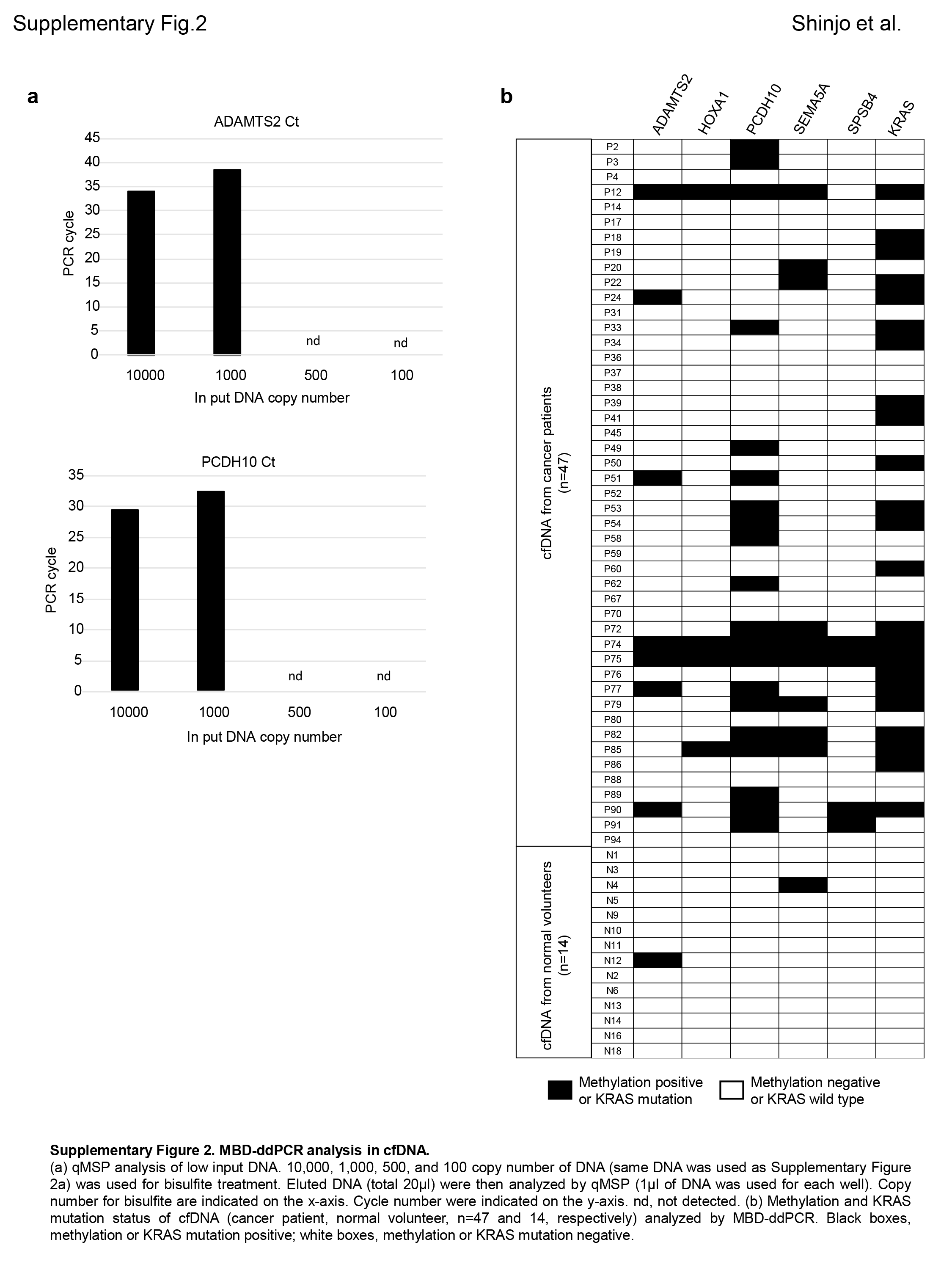

Supplement: S2 Fig — (a) qMSP analysis of low input DNA. 10,000, 1,000, 500, and 100 copy number of DNA (same DNAwas used as S2A Fig) was used for bisulfite treatment. Eluted DNA (total 20pl) were then analyzed by qMSP (1 pl of DNAwas used for each well). Copy number for bisulfite are indicated on the x-axis. Cycle number were indicated on the y-axis. nd, not detected, (b) Methylation and KRAS mutation status of cfDNA (cancer patient, normal volunteer, n = 47 and 14, respectively) analyzed by MBD-ddPCR. Black boxes, methylation or KRAS mutation positive; white boxes, methylation or KRAS mutation negative. (TIF) [file pone.0233782.s008.tif]
